# TEspeX: consensus-specific quantification of transposable element expression preventing biases from exonized fragments

**DOI:** 10.1093/bioinformatics/btac526

**Published:** 2022-07-25

**Authors:** Federico Ansaloni, Nicolò Gualandi, Mauro Esposito, Stefano Gustincich, Remo Sanges

**Affiliations:** Area of Neuroscience, Scuola Internazionale Superiore di Studi Avanzati (SISSA), Trieste 34136, Italy; Central RNA Laboratory, Istituto Italiano di Tecnologia, Genova 16163, Italy; Area of Neuroscience, Scuola Internazionale Superiore di Studi Avanzati (SISSA), Trieste 34136, Italy; Area of Neuroscience, Scuola Internazionale Superiore di Studi Avanzati (SISSA), Trieste 34136, Italy; Central RNA Laboratory, Istituto Italiano di Tecnologia, Genova 16163, Italy; Area of Neuroscience, Scuola Internazionale Superiore di Studi Avanzati (SISSA), Trieste 34136, Italy; Central RNA Laboratory, Istituto Italiano di Tecnologia, Genova 16163, Italy

## Abstract

**Summary:**

Transposable elements (TEs) play key roles in crucial biological pathways. Therefore, several tools enabling the quantification of their expression were recently developed. However, many of the existing tools lack the capability to distinguish between the transcription of autonomously expressed TEs and TE fragments embedded in canonical coding/non-coding non-TE transcripts. Consequently, an apparent change in the expression of a given TE may simply reflect the variation in the expression of the transcripts containing TE-derived sequences. To overcome this issue, we have developed TEspeX, a pipeline for the quantification of TE expression at the consensus level. TEspeX uses Illumina RNA-seq short reads to quantify TE expression avoiding counting reads deriving from inactive TE fragments embedded in canonical transcripts.

**Availability and implementation:**

The tool is implemented in python3, distributed under the GNU General Public License (GPL) and available on Github at https://github.com/fansalon/TEspeX (Zenodo URL: https://doi.org/10.5281/zenodo.6800331).

**Supplementary information:**

Supplementary data are available at *Bioinformatics* online.

## 1 Introduction

Transposable elements (TEs) are repetitive and mobile DNA sequences that occupy large portions of the eukaryotic genomes (Wicker *et al.*, 2007). Although having been considered junk DNA for long time, TEs are now known to play key roles in several biological pathways (Ansaloni *et al.*, 2019; Burns, 2017; Casale *et al.*, 2022; Chuong *et al.*, 2017; Erwin *et al.*, 2014; Floreani *et al.*, 2022; Napoletano *et al.*, 2021; Rodriguez-Terrones and Torres-Padilla, 2018). Therefore, the development of bioinformatics tools enabling the quantification of their expression is a current need. However, the development of such tools is complicated by (i) the repetitive nature of TEs that impairs the unambiguous alignment of RNA-seq reads to specific genomic loci and (ii) the large fractions of exonized TE-derived fragments embedded in canonical transcripts that make it challenging to distinguish between the transcription of autonomously expressed TEs and passive transcription of TE fragments as part of non-TE transcriptional units (Faulkner *et al.*, 2009; Kapusta *et al.*, 2013; Lanciano and Cristofari, 2020). While the former issue could be circumvented either by performing an analysis at the TE consensus level, as implemented in SalmonTE (Jeong *et al.*, 2018), or by using specific statistical algorithms such as the expectation–maximization (EM) used by TEtranscripts (Jin *et al.*, 2015), SQuIRE (Yang *et al.*, 2019), Telescope (Bendall *et al.*, 2019) and L1EM (McKerrow and Fenyö, 2019), the latter still remains mostly unsolved except for a few tools working exclusively on human LINE-1 or ERVs such as L1EM (McKerrow and Fenyö, 2019), TeXP (Navarro *et al.*, 2019) and ERVmap (Tokuyama *et al.*, 2018). An exception should be mentioned also for SQuIRE that attempts to identify the transcript giving rise to the reads quantified at each TE locus. Nevertheless, when using tools that do not directly discriminate between transcription of autonomously expressed TEs and passive transcription of TE fragments, an apparent change in the expression of TEs may simply reflect the variation in the expression of transcripts containing TE-derived sequences (Lanciano and Cristofari, 2020). In order to limit this bias, we have developed TEspeX, a bioinformatics pipeline that quantifies the TE expression at the consensus level without taking into account reads mapping to any annotated non-TE transcript (*n.b.*, canonical transcript sequences do not include introns). TEspeX is a flexible tool, developed to measure the expression of any TE, regardless of its classification.

## 2 Software implementation

**Fig. 1.**
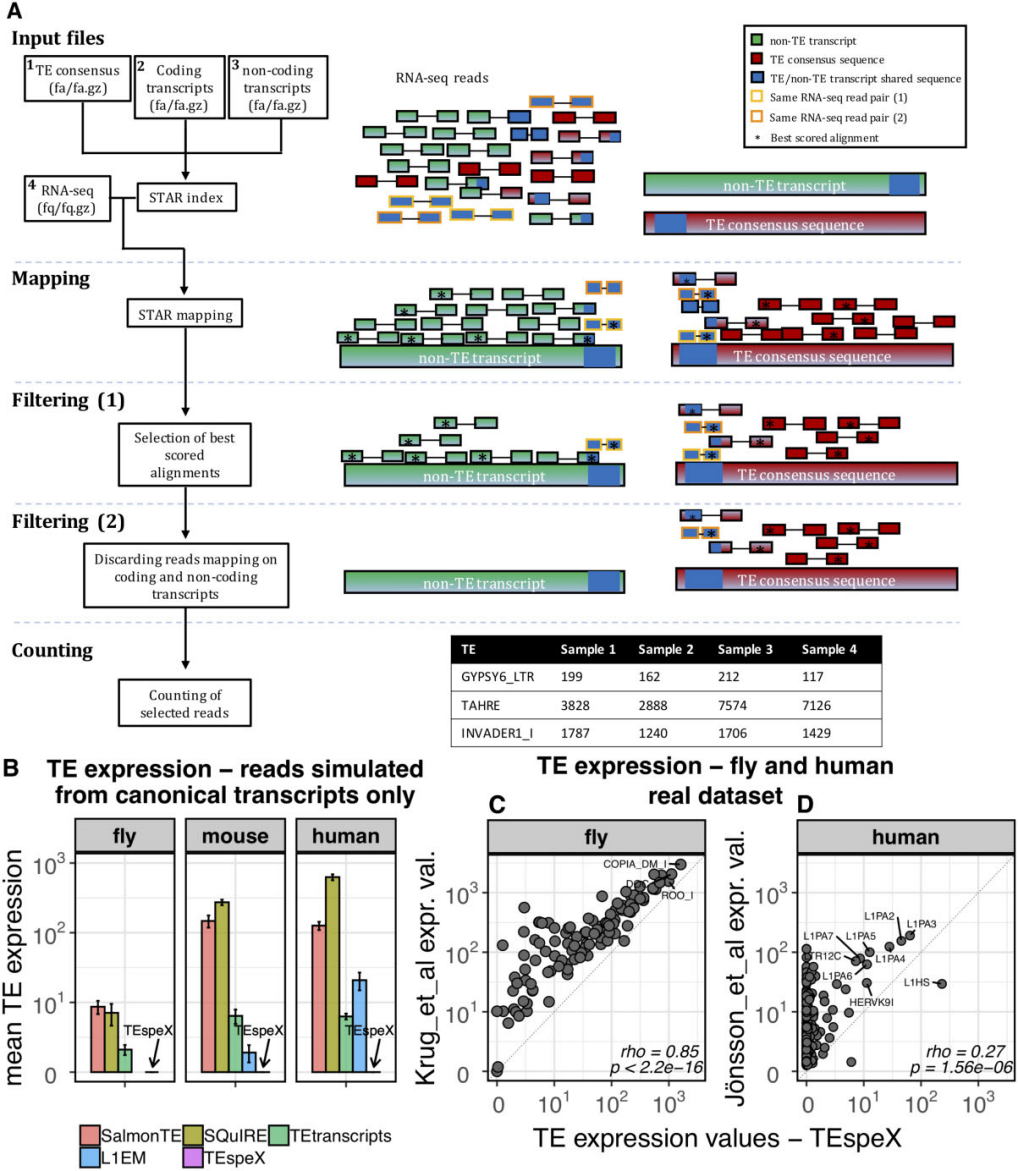
(**A**) Pipeline workflow. Reference transcriptome is generated concatenating TE consensus sequences (1), coding (2) and non-coding transcripts (3). RNA-seq reads (4) are mapped to the reference transcriptome using STAR. Only best scoring alignments are selected and all the reads mapping to any annotated non-TE transcripts are discarded. Selected reads are finally counted. Yellow- and orange-squared RNA-seq read pairs represent two exemplificative examples on TEspeX functioning. Both pairs are aligned to a locus shared between non-TE and TE transcripts. However, while for the orange-squared pair a best alignment to TE sequences can be defined with the read pair therefore considered as TE specific, the yellow-squared one maps with the best score alignment to both non-TE and TE transcripts and it is consequently discarded from the counting. (**B**) Quantification of the TE expression with SalmonTE, SQuiRE, TEtranscripts, L1EM and TEspeX on synthetic RNA-seq reads generated from coding and non-coding transcripts. On *y*-axis, the mean of expression of all the analysed TEs is reported. (**C**) Correlation between TE expression values calculated by Krug and colleagues (*y*-axis) and TEspeX (*x*-axis). (**D**) Correlation between TE expression values calculated by Jönsson and colleagues (*y*-axis) and TEspeX (*x*-axis). In both C and D, expression levels are reported as mean of expression calculated among all the samples of each dataset

TEspeX is developed in python3 and takes advantage of STAR (v2.6.0c) (Dobin *et al.*, 2013), samtools (v1.3.1) (Li and Durbin, 2009) and Picard (v2.18.4) (https://broadinstitute.github.io/picard/). The pipeline rationale is to select for the TE expression quantification of only the RNA-seq reads deriving from the transcription of autonomously expressed TEs. Therefore, all the reads possibly transcribed from TE fragments embedded in canonical coding/non-coding non-TE transcripts are discarded. To this end, first, the reference transcriptome composed by TE consensus sequences and annotated coding/non-coding transcripts (introns not included) is generated and indexed, then RNA-seq reads are mapped to the reference transcriptome. Best scoring alignments are selected and all the reads mapping to any annotated non-TE transcript are discarded. Finally, the selected reads are counted (Fig. 1A). Comprehensive description of the pipeline implementation is reported in Supplementary Data Note S1.

## 3 Validation

To test the capability of TEspeX in quantifying TE expression in Metazoan, we generated *in silico* RNA-seq reads from the Repbase TE consensus sequences (Bao *et al.*, 2015) of *Caenorhabditis elegans*, *Drosophila melanogaster*, *Danio rerio*, *Mus musculus* and *Homo sapiens* (see methods used in Supplementary Data Note S2). This approach allowed to generate a known number of reads from each TE consensus, assigning a known expression value to each TE. Then, to test the concordance between the known TE expression levels and the ones calculated by TEspeX, the TE expression was calculated using TEspeX and compared with the known counts, in each analysed species. Our results showed that the TE expression levels measured by TEspeX were significantly correlated with the number of artificial reads generated *in silico*, in all the analysed species (Spearman’s rho >0.96, *P*-value < 2.2*e*−16), supporting the evidence of TEspeX properly working on a broad set of species (Supplementary Data Note S2). Then, to test TEspeX capability in quantifying TE expression without taking into consideration reads transcribed as part of non-TE transcripts, we generated artificial RNA-seq reads from canonical non-TE transcripts of *D.melanogaster* (dm6), *M.musculus* (mm39) and *H.sapiens* (hg38). TE expression was then quantified at the consensus sequence level using TEspeX, SalmonTE (Jeong *et al.*, 2018), SQuIRE (Yang *et al.*, 2019), TEtranscripts (Jin *et al.*, 2015) and L1EM (for murine and human LINE-1 only), a tool specifically developed to quantify reads deriving from LINE-1 autonomous transcription (McKerrow and Fenyö, 2019). Given that no RNA-seq reads were generated from TE consensus sequences, the expression of TEs from this analysis was expected to be null. However, the results showed that all the tools, except TEspeX, assigned some expression levels to TEs (Fig. 1B and Supplementary Data Note S3). The reads at the basis of these results derived from TE fragments embedded in non-TE transcripts and should not be considered as deriving from TE transcription, as correctly shown by TEspeX. We then tested if the TE expression measured was mainly deriving from TEs embedded in 3′ UTRs, repeating the analysis on the same synthetic RNA-seq dataset after the removal of all the reads mapping to transcript 3′ UTRs. Although we noticed a general decrease in the expression levels, all the tested tools except TEspeX assigned expression levels to TEs, thus suggesting that the detected signal does not exclusively derive from TE fragments embedded in 3′ UTRs (Farré *et al.*, 2016; Supplementary Data Note S4).

Having assessed the proper functioning of TEspeX on *in silico* generated data, we next tested the tool on real RNA-seq publicly available datasets from *D.melanogaster* (Krug *et al.*, 2017) and *H.sapiens* (Jönsson *et al.*, 2019). In the *Drosophila* dataset, Krug and colleagues observed transcriptional activation of TEs upon the over-expression of wild-type human TDP-43 in neuronal and glial cells. To test our tool on the same dataset, TE expression levels were calculated by using TEspeX. Quantification of TE expression levels by TEspeX highlighted a significant correlation between the expression levels calculated by the authors of the article and those calculated by TEspeX (rho = 0.85, *P*-value < 2.2*e*−16) (Fig. 1C and Supplementary Data Note S5). Next, to compare the TE expression levels measured by TEspeX with the ones measured by other standalone pipelines, the TE expression levels in the same dataset were calculated by SalmonTE, SQuIRE and TEtranscripts and correlated with those calculated by TEspeX. The results highlighted high concordance among all the tested tools and, in particular, the expression levels calculated by TEspeX resulted significantly correlated with those calculated by all the other tested tools (rho > 0.80, *P*-value < 2.2e−16 in all the comparisons) (Supplementary Data Note S5). Moreover, upon the identification of the differentially expressed TEs, the TEspeX quantifications recapitulated those from the authors confirming that (i) TDP-43 over expression in glia and neurons induced up-regulation of TEs, (ii) TEs involved in such process were almost exclusively retrotransposons (LINE and LTR) and (iii) gypsy retrotransposon resulted among the top upregulated TEs, exclusively in the glia (Supplementary Data Note S6).

Then, the capacity of TEspeX in quantifying TE expression levels in real RNA-seq data was tested on a *H.sapiens* dataset where TE transcriptional activation was observed in human neural progenitor cells (hNPC) upon DNMT1 knock-out (KO) by Jönsson *et al.,* (2019). Quantification of the TE expression levels with TEspeX highlighted a significant positive correlation between the expression levels calculated by the authors and those calculated by TEspeX (rho = 0.27, *P*-value = 1.56e−06) (Fig. 1D and Supplementary Data Note S7). Although the TE expression levels calculated by Jönsson *et al.,* (2019) and the ones calculated by TEspeX resulted significantly correlated (*P*-value = 1.56e−06), the correlation coefficient was low (rho = 0.27), with several TEs identified as expressed by the custom pipeline used by Jönsson *et al.,* (2019) and not identified to be expressed by TEspeX. The same was observed when comparing the TE expression levels measured by TEspeX with the ones calculated by other standalone pipelines such as SalmonTE, SQuIRE and TEtranscripts (Supplementary Data Note S7). This could be the consequence of the filtering that TEspeX applies in order to discard potential false positive reads possibly deriving from non-TE transcripts and not applied by SQuIRE, SalmonTE or TEtranscripts. This hypothesis was confirmed by the evidence that the tool which better correlated with TEspeX was L1EM (rho = 0.94, *P* < 2.2e−16), which is the only tool, in addition to TEspeX, implemented to specifically distinguish between autonomous and passive TE transcription (Supplementary Data Note S7). We then wondered whether the TEspeX analysis could recapitulate the results previously validated, through both computational and experimental approaches, by Jönsson *et al.,* (2019). Upon the identification of the differentially expressed TEs following the DNMT1 KO in hNPC, the up-regulation of young LINE-1 elements (L1HS, L1PA2 and L1PA3 subfamilies) as well as of the LTR subfamily LTR12C was highlighted by the TEspeX analysis. Therefore, the TEspeX analysis results recapitulated all the results previously observed by Jönsson *et al.,* (2019) (Supplementary Data Note S8).

An additional source of TE passive expression is represented by the presence of introns in the mRNA preps. It is now clear that the total RNA RNA-seq libraries and, to a lesser extent, the polyA+ ones might contain intronic sequences deriving from immature transcripts (Deininger *et al.*, 2017; Zaghlool *et al.*, 2013). This bias appears to particularly affect nuclear RNA samples whereas cytoplasmic preparations largely, but not completely, reduce intron contaminations (Zaghlool *et al.*, 2013). Given that TEs have the tendency to localize within intronic regions (Medstrand *et al.*, 2002), sequencing reads originating from unprocessed introns can be erroneously detected as proper TE expression by TE expression quantification tools (Deininger *et al.*, 2017; Gualandi *et al.*, 2022). Considering the above, although TEspeX has not been benchmarked on datasets deriving from different library preparations, polyA+ cytoplasmic RNA-seq libraries should be preferred when measuring TE expression with TEspeX. Another potential bias also pertaining to this issue might be represented by intron retention in mature transcripts. To avoid bias in the TE expression quantification deriving from this phenomenon, we propose to identify the retained introns before running TEspeX and to add the identified retained introns to the TEspeX masking library (–mask parameter). In this way, TEspeX will not take into account reads transcribed from the retained introns. In Supplementary Data Note S9, an example on how to perform a correction to avoid the intron retention bias is reported and it is demonstrated that, at least in the polyA+ RNA-seq dataset from Jönsson *et al.* (2019), intronic reads do not impact the final results. The performed correction, however, can result useful in cases in which differential intron retention, or any bias given by intronic reads, is present in the analysed dataset.

Next, we reasoned that if the expression measured by TEspeX is really derived from autonomously expressed TEs, the expression of evolutionary ancient TEs should be null, or very low, as evolutionary ancient TEs are more likely to have been transcriptionally silenced across the evolution. To test this hypothesis, first we stratified *Drosophila* and *H. sapiens* TEs based on their evolutionary age and genomic location. Second, we selected the 10 youngest and 10 oldest intergenic and intronic TEs in both species and, third, we quantified their expression levels by using TEsepX in the Krug and Jönsson RNA-seq datasets previously analysed (see methods used in Supplementary Data Note S10). Remarkably, our results showed that while the young TEs resulted as expressed, the ancient TEs were overall not detected to be expressed, considering both the intergenic and the intronic elements, in both *Drosophila* and *H. sapiens* (Supplementary Data Note S10).

## 4 Conclusions

TEspeX is a pipeline developed to quantify the TE expression at the consensus level. The tests performed on artificial and publicly available RNA-seq datasets confirmed the functioning of the tool in preventing to call false positive TE expression using reads potentially deriving from TE fragments embedded in non-TE transcripts. Clearly, in case of reads whose sequence is identical between an autonomously expressed TE and a TE fragment embedded in a non-TE transcript, the tool cannot perform a proper assignment. TEspeX will therefore discard such reads, thus contributing to the production of false negative results. This particularly affects human Alu, which are known to have been frequently exonized across the evolution (Schmitz and Brosius, 2011) and that are characterized by many short and highly similar genomic sequences (Supplementary Data Notes S11 and S12). However, as already discussed by Ewing (2015), when analysing TEs, it is hard to develop and tune methods characterized by high sensitivity while maintaining high specificity (Ewing, 2015). This is due to the repetitive nature of TEs and to the fact that portions of their sequences have frequently been exapted during evolution. Ewing (2015) also suggests that an ensemble approach, combining different methods, may be the solution to increase the sensitivity when analysing TEs using short reads. Within this context, TEspeX fills an important gap among the different approaches currently available for the analysis of TE expression.

## Funding

This work was supported by the International School for Advanced Studies (SISSA) PhD fellowship and Istituto Italiano di Tecnologia (IIT) postdoctoral fellowship to F.A. and the International School for Advanced Studies (SISSA) PhD fellowships to N.G. and M.E.

*Conflict of Interest*: none declared.

## Supplementary Material

btac526_Supplementary_Data
