# Supplementary material for: TEspeX: consensus-specific quantification of transposable element expression preventing biases from exonized fragments
Source: Bioinformatics. 2022 Jul 25;38(18):4430–3. doi: 10.1093/bioinformatics/btac526 (PMC9477521; doi:10.1093/bioinformatics/btac526)
Supplement: btac526_Supplementary_Data [file btac526_supplementary_data.pdf]

<u>**SUPPLEMENTARY DATA**</u>

TEspeX: consensus-specific quantification of transposable element expression preventing biases from exonized fragments

Ansaloni F.[1,2], Gualandi N.[1], Esposito M.[1], Gustincich S.[2*] and Sanges R.[1,2*]

1 Area of Neuroscience, International School for Advanced Studies (SISSA), Trieste, 34136, Italy.

2 Central RNA Laboratory, Istituto Italiano di Tecnologia, Genova, 16163, Italy.

* Co-corresponding authors

Supplementary Data Note 1

Software implementation and recommendations

TEspeX requires as input files short Illumina RNA-seq reads in FASTQ format as well as three FASTA files containing annotated coding transcripts, non-coding transcripts and TE consensus sequences. Although we recommend to download the coding/non-coding FASTA files from Ensembl (Zerbino *et al.*, 2018) and the TE consensus sequences from the Dfam (Hubley *et al.*, 2016) or Repbase (Bao *et al.*, 2015) databases, TEspeX is implemented to run on FASTA files potentially retrieved from any source of annotation. Following our recommendations, a help page illustrating how to correctly query the Dfam and Repbase database is available here: https://github.com/fansalon/TEconsensus.

The first step performed by TEspeX is to build the reference transcriptome by merging the three input FASTA files. The transcriptome index is, then, generated by STAR (Dobin *et al.*, 2013) *(--runMode genomeGenerate*) using default parameters except for *--genomeSAindexNbases* and *--genomeChrBinNBits* that are *de novo* calculated to avoid segmentation fault errors based on the length and number of sequences contained in the newly generated transcriptome (as suggested by the STAR manual). Then, RNA-seq reads are mapped to the reference transcriptome using STAR with default parameters except for the maximum number of mismatches allowed for each alignment (set to 4% of the alignment length *--outFilterMismatchNoverLmax 0.04*) and the assignment of the primary flag that is attributed to all the alignments with the best alignment score (AS) (*--outSAMprimaryFlag AllBestScore*). Overall, the combination of these STAR parameters allows to handle the multimapping reads as follow:

1. for a given read/read pair, only the alignments scoring an AS comprised between the maximum AS of the given read/read pair ($\boldsymbol{AS_{max}}$) and $\boldsymbol{AS_{max}}$-1 are selected (STAR default *--outFilterMultimapScoreRange 1*)

2. for each read/read pair, the allowed maximum number of alignments selected in "1." is set to 10 (STAR default *--outFilterMultimapNmax 10*). If more than 10, all the alignments of a given read/read pair are discarded.
3. the primary alignment flag is assigned to all the selected alignments showing the maximum AS (*--outSAMprimaryFlag AllBestScore*)

To avoid counting reads aligning to TE fragments embedded in non-TE transcripts, only the alignments flagged as primary (*i.e.,* the best alignments) are selected by using samtools (Li *et al.*, 2009). Then, reads mapping with best AS to any annotated non-TE transcript are discarded. Finally, the selected reads, mapping with best AS exclusively on TEs and in the proper orientation (in case the RNA-seq library is strand specific), are counted. Once completed, TEspeX outputs a table reporting the number of reads mapped to each TE consensus analysed (*i.e.*, TE expression levels), three BAM files (all the alignments, best alignments only and TE-specific alignments only), a log file and a summary file containing the mapping statistics. The number of computing threads to be used can be defined by the user and, when a multi-node HPC systems controlled by SLURM is available, TEspeX can run on multiple computing nodes

In cases in which novel non-annotated transcripts containing exonized TE fragments are transcribed in a given sample, the RNA-seq reads deriving from these transcripts are not filtered-out by TEspex. If the user is aware of this situation manifesting in the sample under analysis, the FASTA sequences of the non-annotated transcripts can be provided to TEspeX through the *--mask* parameter. These sequences will be considered by TEspeX as part of the coding/non-coding transcriptome and the reads mapping to them with best AS will be discarded according to the TEspeX workflow. If the user is not aware of any novel non-annotated transcript but prefer to run a more stringent analysis, our suggestion is to run a *de-novo* assembly of the transcriptome using the RNAseq data and use the *--mask* parameter with the generated assembly after the removal of the assembled full-length autonomous TEs.

Considerations on multimapping reads

Like many tools TEspeX does not solve the problem of multimapping reads and relies on thresholds set by the user. Tools like SQuIRE and TEtranscripts, exploiting the expectation-maximization (EM) algorithm for multimapping handling and that map reads to the genome, set and recommend the number of multimapping threshold to 100. Since TEspeX is implemented to map reads to a reference transcriptome composed only by TE consensus plus canonical coding/non-coding transcripts a lower threshold is used and suggested. We calculated the number of multimapping that are retained by TEspeX in two RNA-seq dataset that we used in this manuscript to test/validate our pipeline (Jönsson *et al.*, 2019; Krug *et al.*, 2017). In the Jönsson *et al.* human RNA-seq dataset, there are only 1% of reads showing more than 5 identical multimappings. Instead, in the Drosophila dataset from Krug *et al.* there are 11% of reads mapping to more than 5 sequences with the best alignment score. This highlights how the number of multimapping depends also on the analysed species. For this reason, starting from TEspeX v1.2.0 we give the user the possibility to change this threshold through the "*--multimap*" parameter. Through this parameter the user can set the maximum number of alignments (with alignment score >= $\boldsymbol{AS_{max}}$-1) allowed for each read in order not to discard the read. Retaining only uniquely mapped reads is generally too stringent, as it is common that different TE consensus sequences share large portions of nucleotide sequences. For example, in mouse there are 7 members of the L1MdA group and their consensus share to up more than 97% of nucleotide sequence. Retaining only uniquely mapped reads would mean to lose most of the reads deriving from the expression of these elements. Manually inspecting the TEs consensus database of different species, we observed that rarely, if ever, more than 10 consensus sequences representing slightly different version of the same element exist. We therefore reasoned to set to 10 by default the maximum number of multimapping.

Supplementary Data Note 2

TEspeX capability in quantifying TE expression from *in silico* simulated data in different Metazoan species

To test the capability of TEspeX in quantifying the expression of TEs in different species, four replicates of 125x2 paired-end reads were generated *in silico* from Repbase TE consensus sequences of *Caenorhabditis Elegans*, *Drosophila melanogaster*, *Danio rerio*, *Mus musculus* and *Homo sapiens* by using the polyester (Frazee *et al.*, 2015) and the BBmap reformat tools (Bushnell, 2014). The number of reads generated for each TE consensus depends on the consensus length with 15 reads generated every kilobase of consensus. Then, the TE expression levels in each species were calculated by using TEspeX (--*strand yes* parameter) and the TEspeX counts compared with the counts of the generated simulated ones. To this end, Spearman correlation has been used to calculate Spearman's rank correlation coefficient (rho) and the statistical significance value. Results indicate that TEspeX quantifications correctly reflect the number of generated simulated reads showing Spearman's rho values higher than 0.96 and p-value < 2.2e-16, in all the analysed species (**Supplementary Fig. S1**).

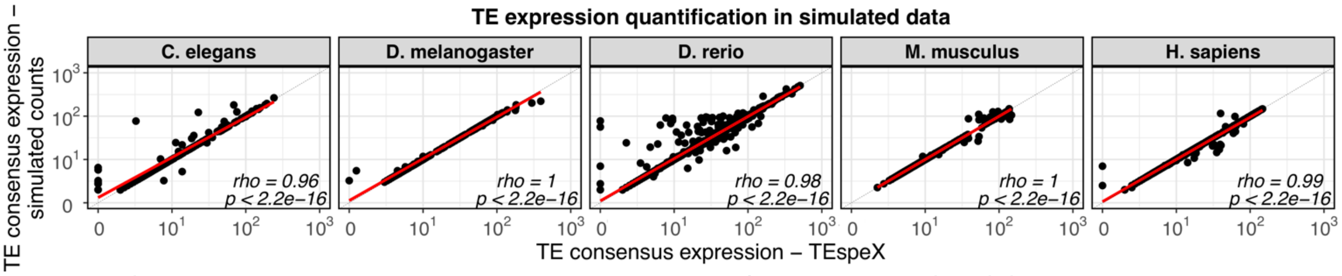

Supplementary Figure S1:TEspeX TE expression quantification in simulated data
Scatter plots reporting the TE expression as calculated by TEspeX (x-axis) and the number of counts of *in silico* generated reads from each TE (y-axis). Each dot represents the mean expression level of each TE among the 4 different replicates. Note the log10 scale. Correlation coefficients were calculated by using Spearman correlation.

<u>**Note**</u>: in all the correlation analyses performed in this manuscript, Spearman correlation rather than Pearson has been used, as Pearson correlation assumes: i) normality of the data (which is not always the case) and ii) absence of outliers (Pearson correlation is highly sensitive to the presence of outliers, whereas Spearman is not).

Supplementary Data Note 3

Testing TEspeX capability in quantifying TE expression filtering out reads transcribed as part of non-TE transcripts

To test TEspeX capability in quantifying TE expression without taking into consideration reads transcribed as part of non-TE transcripts, 125x2 paired-end RNA-seq reads from *Drosophila melanogaster* (dm6), *Mus musculus* (mm39) and *Homo sapiens* (hg38) canonical transcripts (coding and non-coding) were generated *in silico* using the tools listed in **Supplementary Data Note 2**. TE expression levels have then been calculated at the consensus level using SalmonTE (Jeong *et al.*, 2018), SQuIRE (Yang *et al.*, 2019), TEtranscripts (Jin *et al.*, 2015), L1EM (McKerrow and Fenyö, 2019) and TEspeX.

Parameters used for each analysis are described below:

- **SalmonTE**: 'quant'algorithm has been used, expression levels were measured as raw counts (*--exprtype counts*) and SalmonTE manually curated TE fasta sequences were used for each species ('dm' for *D. Melanogaster*, 'mm' for *M. musculus* and 'hs' for *H. Sapiens*)
- **SQuIRE**: 'argument.sh' file has been filled-in with the required input files, setting 'strandedness=2' and using the dm6, mm39 and hg38 genome builds for *D. Melanogaster*, *M. musculus* and *H. Sapiens*, respectively. TE expression of each TE subfamily (consensus level) has been considered in this analysis (*subFcounts.txt files)
- **TEtranscripts**: reads were mapped to the dm6, mm39 and hg38 reference genomes downloaded from ensembl/gencode using STAR (Dobin *et al.*, 2013) with default parameters except for *--outFilterMultimapNmax* and *--winAnchorMultimapNmax* parameters that were set to 100, as suggested by the TEtranscripts developers (https://github.com/mhammell-laboratory/TEtranscripts). TEcount algorithm has then been used to quantify TE expression levels providing as gtf file the ensembl/gencode gtf, as 'TE file" the gtf TE annotation file retrieved directly from Molly Hammel lab's website

(http://labshare.cshl.edu/shares/mhammelllab/www-data/TEtranscripts/TE_GTF/) and setting the strand parameter to 'forward'

- **L1EM**: only murine and human LINE-1 expression has been calculated as currently the tool is implemented to quantify the expression of these elements only. BAM alignment files were generated by mapping the RNA-seq reads to the mm39 and hg38 reference genomes using bwa mem (Li and Durbin, 2009) with default parameters. Next, the run_L1EM_mm39.sh and run_L1EM_withlessmemory.sh tools were run for mouse and human, respectively. L1EM outputs the LINE-1 expression at the single locus level. Therefore, in order to obtain a quantification at the consensus level, expression levels of single LINE-1 loci belonging to the same LINE-1 subfamily were summed. Only the '*Only*' count estimations were considered in this calculation (includes only the annotated element, supported by sense reads that fall entirely within the LINE-1 element)
- **TEspeX**: coding and non-coding transcripts were retrieved from ensembl/gencode whereas TE consensus sequences from Repbase. Default parameters were used setting the --strand parameter to 'yes'.

Results from this analysis are described in the Main text of the article and depicted in **Fig. 1B**.

Please note that both L1EM and SQuIRE could be used in ways that would increase specificity. For instance, when using L1EM, any locus with less than 100 reads mapped can be dropped as these are often considered false positives. For SQuIRE, any transcript that starts more than 25-50 bases upstream of the annotated TE could be discarded.

Supplementary Data Note 4

Testing if TEs expression detected on the *in silico* RNA-seq reads from canonical coding and non-coding transcripts originates mainly from 3' UTRs

Alu elements are known to be enriched in human transcript 3' UTRs (Farré *et al.*, 2016). Therefore, we wondered whether the TE expression levels detected by SalmonTE, SQuIRE, TEtranscripts and L1EM on *in silico* simulated RNA-seq reads from canonical coding and non-coding transcripts (see **Supplementary Data Note 3** and **Fig. 1B**) might derive from TEs embedded in the 3' UTRs of annotated transcripts. To this end, the RNA-seq reads *in silico* simulated from *Drosophila*, mouse and human transcripts (see **Supplementary Data Note 3**), were mapped to the respective reference genomes. Then, sequencing reads mapping to any annotated 3' UTR region were discarded, thus obtaining a set of sequencing reads depleted in 3' UTR reads. The new RNA-seq datasets were then used to quantify again the TE expression levels by using SalmonTE, SQuIRE, TEtranscripts, L1EM and TEspeX following the same workflow described in **Supplementary Data Note 3**. The results showed that, even when discarding sequencing reads mapping in 3' UTRs, all the tested tools, except TEspeX, did assign some level of expression to the TEs (**Supplementary Fig. S2A** and **B**). This suggests that not all the TE expression detected by the tested tools originates from TEs embedded in transcript 3' UTRs, but also from TE fragments embedded in other portions of the transcript body.

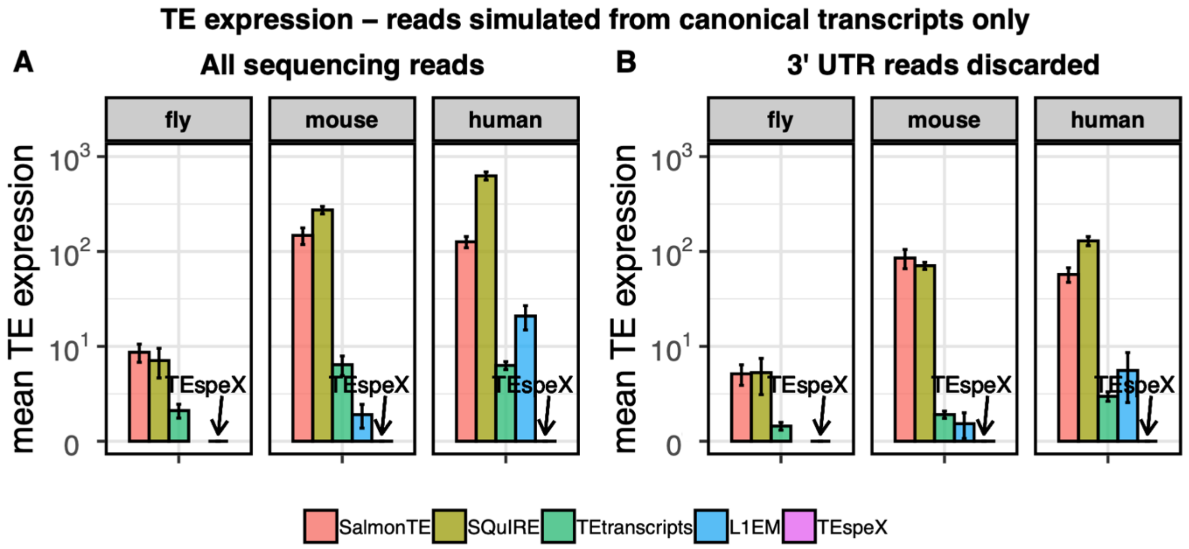

Supplementary Figure S2: TEspeX TE expression quantification in simulated data depleted from 3' UTR reads

(**A**) Quantification of the TE expression with SalmonTE, SQuiRE, TEtranscripts, L1EM and TEspeX on synthetic RNA-seq reads generated exclusively from coding and non-coding transcripts. On y-axis, the mean of expression of all the analysed TEs is reported. This is the same plot represented in **Fig. 1B** of the main text. (**B**) Same as in (**A**), but TE expression levels were calculated on RNA-seq data were 3' UTR-mapped reads have been discarded.

Supplementary Data Note 5

Comparison among tools in calculating TE expression values in a publicly available *Drosophila* RNA-seq dataset

To test TEspeX capability in measuring TE expression levels from a real RNA-seq dataset and to compare the TE expression levels measured by TEspeX with the TE expression calculated by different standalone pipelines, RNA-seq data were retrieved from a recent publication (Krug *et al.*, 2017). TE expression values were measured at the consensus level by using TEspeX as well as SalmonTE, TEtranscripts and SQuIRE. The tools were run with the same parameters described in **Supplementary Data Note 3**. The strandedness of the library was set to “yes”, “forward” and “2” for TEspeX, TEtranscripts and SQuIRE, respectively. TE expression values as calculated by Krug and colleagues (by using a previous version of TEtranscripts) were directly retrieved from the supplementary table S2 and S3 of the original manuscript. Correlation coefficients and relative statistical significance values were measured by Spearman correlations, as described in **Supplementary Data Note 2**.

Our results highlighted high concordance among all the tested tools and, in particular, the expression levels calculated by TEspeX resulted significantly correlated with those calculated by all the other tested tools (rho > 0.80 and p-value < 2.2e-16 in all the comparisons) (**Supplementary Fig. S3** and **Supplementary Fig. S4**). The tool that shows the lowest correlation with TEspeX is SQuIRE (rho=0.80, p-value < 2.2e-16), probably as a consequence of the different approaches implemented by the two tools (TEspeX is implemented to work on TE consensus, whereas SQuIRE on single loci). On the other hand, the tool that correlates the best with TEspeX is SalmonTE (rho=0.89, p-value < 2.2e-16). Notably, these are the only two tools that map the sequencing reads to the TE consensus sequence.

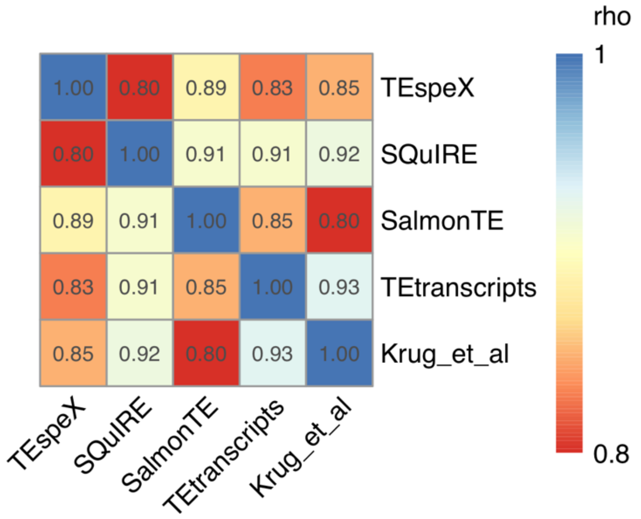

Supplementary Figure S3: Spearman's rank correlation coefficients among all the tested tools.
TE expression levels were calculated in a *Drosophila* publicly available RNA-seq dataset (Krug *et al.*, 2017) by using TEspeX, SQuIRE, SalmonTE, TEtranscripts and retrieving the counts as calculated by the authors of the article. Spearman correlation has been used to calculate the degree of concordance among the different tested tools in measuring the TE expression values.

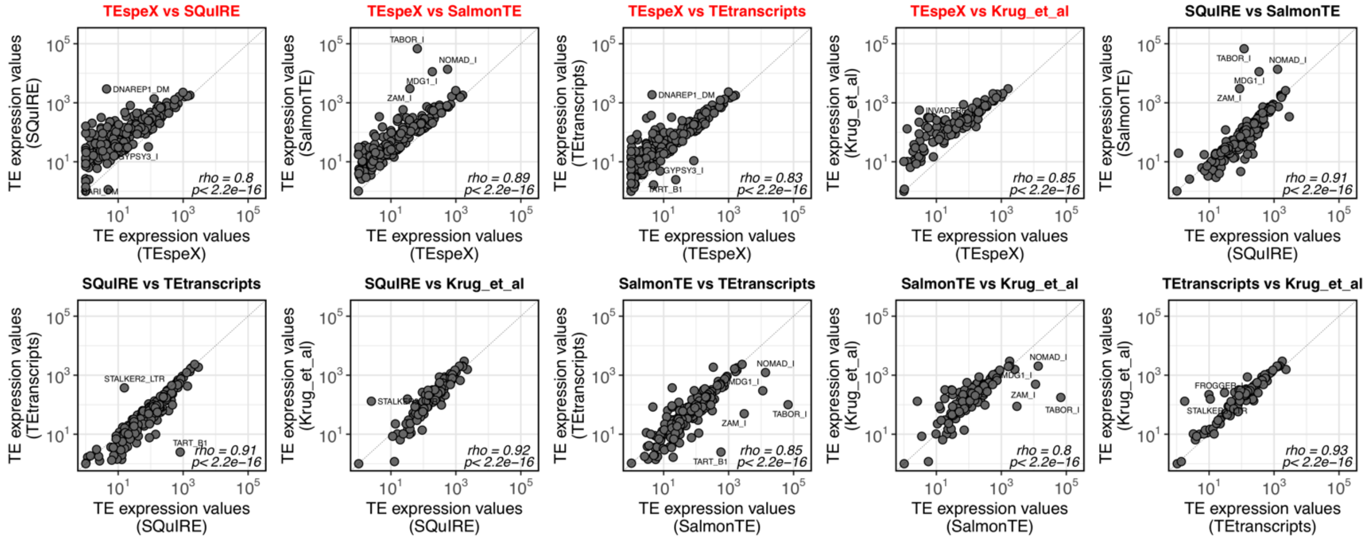

Supplementary Figure S4: comparison among different TE expression quantification standalone pipelines
Scatter plots reporting the TE expression levels calculated by the different tools tested. Each dot represents the mean TE expression levels calculated among the entire Krug *et al.* dataset. Spearman's rank correlation coefficients (rho) and p-values are reported for each comparison. Red-colored titles indicated comparisons involving TEspeX.

Supplementary Data Note 6

Differentially expressed TEs in the Krug *et al.* dataset – TEspeX analysis

Differentially expressed TEs in glial and neuronal cells upon the over-expression of human TDP-43 were identified by using edgeR (Robinson *et al.*, 2010). EdgeR normalisation of raw read counts has been applied using the TMM method providing as library size the total number of reads mapped to the reference genome in each sample. Common, trended and tagwise dispersions were estimated by maximizing the negative binomial likelihood (default). Next, differentially expressed TEs were identified for each pairwise comparison performing a quasi-likelihood F-tests (glmQLFit and glmQLTest). TEs were selected as differentially expressed when showing FDR < 0.05 and log2FC < -0.58 or > 0.58 (1.5-fold in linear scale).

Our results (**Supplementary Fig. S5**) highlighted that, as already discussed in the main text, the TEspeX quantifications recapitulated those from the authors of the article (Krug *et al.*, 2017) confirming that:

1. TDP-43 over expression in glia and neurons induces up-regulation of TEs
2. TEs involved in such process are almost exclusively retrotransposons (LINE and LTR)
3. *gypsy* (GYPSY_I) retrotransposon results among the top upregulated TEs exclusively in the glia

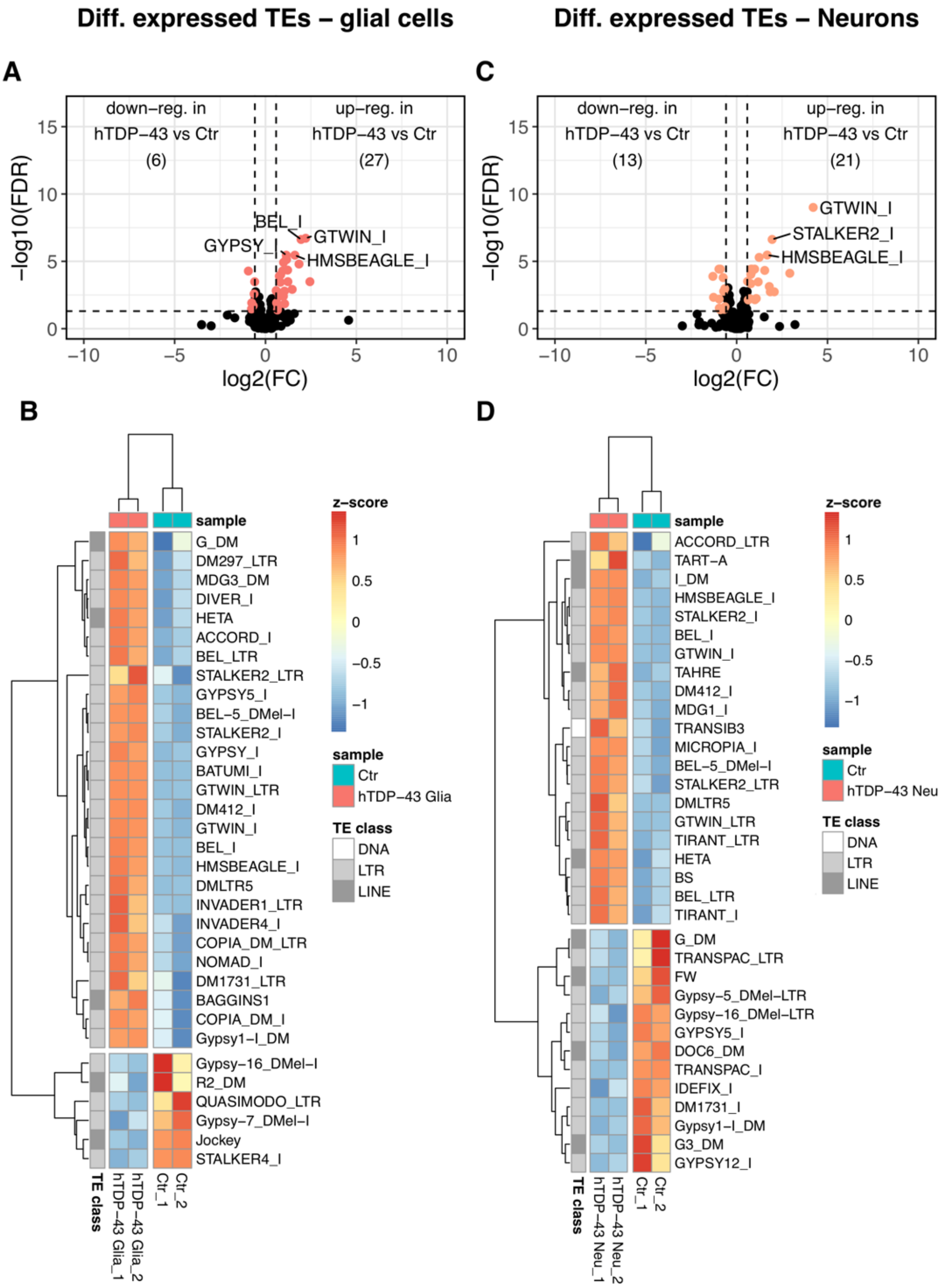

Supplementary Figure S5: differentially expressed TEs in glia and neurons upon hTDP-43 overexpression.
(A) Volcano plot reporting the differentially expressed TEs in glia upon the overexpression of hTDP-43. Consistently with Krug et al. results, GYPSY_I (referred as *gypsy* in Krug et al.) resulted among the most significantly up-regulated TEs. **(B)** Heatmap showing the differentially expressed TE expression levels. Grey-scaled bar on the left of the heatmap shows the TE class each DE TE belongs to. Only retrotransposons (LTR and LINE) resulted differentially expressed in this condition, with no DNA transposon resulting significantly de-regulated. **(C)** Volcano plot reporting the differentially expressed TEs in neurons upon the overexpression of hTDP-43. **(D)** Heatmap showing the differentially expressed TE expression levels. As observed in glia, the large majority of the DE TEs are classified as retrotransposons (LTR and LINE) with only one DNA transposon resulting significantly de-regulated (TRANSIB3).

Supplementary Data Note 7

Comparison among tools in calculating TE expression values in a publicly available *H. sapiens* RNA-seq dataset

TEspeX was then tested on RNA-seq data retrieved from a recent publication where the expression of TEs was observed in human neural precursor cells (hNPC) upon DNMT1 knock-out (KO) (Jönsson *et al.*, 2019). In their work Jönsson and colleagues measured the TE expression values at the single locus by using a custom pipeline that: i) discards all the multi-mapping reads and ii) compute the expression levels of all the TE single loci annotated in RepeatMasker and non-overlapping NCBI gene exons. TE expression values as calculated by Jönsson and colleagues were directly retrieved from https://www.ncbi.nlm.nih.gov/geo/query/acc.cgi?acc=GSE107580. In order to obtain a quantification at the consensus level, expression values of single TE loci belonging to the same TE subfamily were summed. TE expression values were then calculated also by using SalmonTE, TEtranscripts, SQuIRE and L1EM as described in **Supplementary Data Note 3**. The strandedness of the library was set to "reverse" for both TEspeX and TEtranscripts and to "1" for SQuIRE. Correlation coefficients and relative statistical significance values were measured by Spearman correlations, as described in **Supplementary Data Note 2**.

Although our results highlighted a significant correlation between the TE expression levels calculated by TEspeX and the ones calculated by the authors of the article (p-value = 1.56e-06), the correlation coefficient was low (rho=0.27) with several TEs resulting expressed according to the custom pipeline used by the author of the article but not to TEspeX (**Supplementary Fig. S6** and **Supplementary Fig. S7**). This discrepancy might be the consequence of the filtering that TEspeX performs in order to discard false positive reads deriving from TE fragments embedded in non-TE transcripts. This correction is indeed not implemented in the custom pipeline developed by the authors of the article that, while discarding from their analysis TEs annotated

in overlap with any annotated genes, do not discard the sequencing reads potentially deriving from these loci. It is not clear how the authors counted the reads assigned to each TE and, most importantly, whether only reads mapping in the proper orientation were considered. Similarly, when comparing TEspeX with SQuIRE, SalmonTE and TEtranscripts the correlation scores, although statistically significant, were rather low (rho=0.19, 0.67 and 0.22, respectively) with several TEs not resulting expressed according to TEspeX while showing expression according to the three aforementioned tools (**Supplementary Fig. S6** and **Supplementary Fig. S7**). This, once again, could be the consequence of the filtering implemented in TEspeX to discard reads possibly deriving from non-TE transcripts and not applied by SQuIRE, SalmonTE and TEtranscripts. This hypothesis is indeed strengthened by the evidence that the tool that better correlates with TEspeX is L1EM (and viceversa) (rho=0.94, $p < 2.2e-16$), which is the only tool, in addition to TEspeX, implemented to distinguish between autonomous and passive transcription (**Supplementary Fig. S6** and **Supplementary Fig. S7**). However, while L1EM is implemented to quantify the expression of exclusively young LINE-1 elements of mouse and human, TEspeX is developed to measure the expression levels of any type of TEs, in potentially any species.

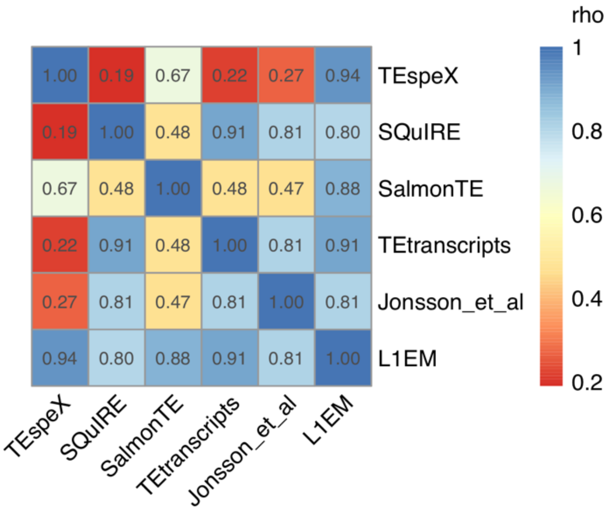

Supplementary Figure S6: Spearman's rank correlation coefficients among all the tested tools. TE expression levels were calculated in a human publicly available RNA-seq dataset (Jönsson et al., 2019) by using TEspeX, SQuIRE, SalmonTE, TEtranscripts, L1EM and retrieving the counts as calculated by the authors of the paper. Spearman correlation has been used to calculate the degree of concordance among the tested tools in measuring the TE expression values.

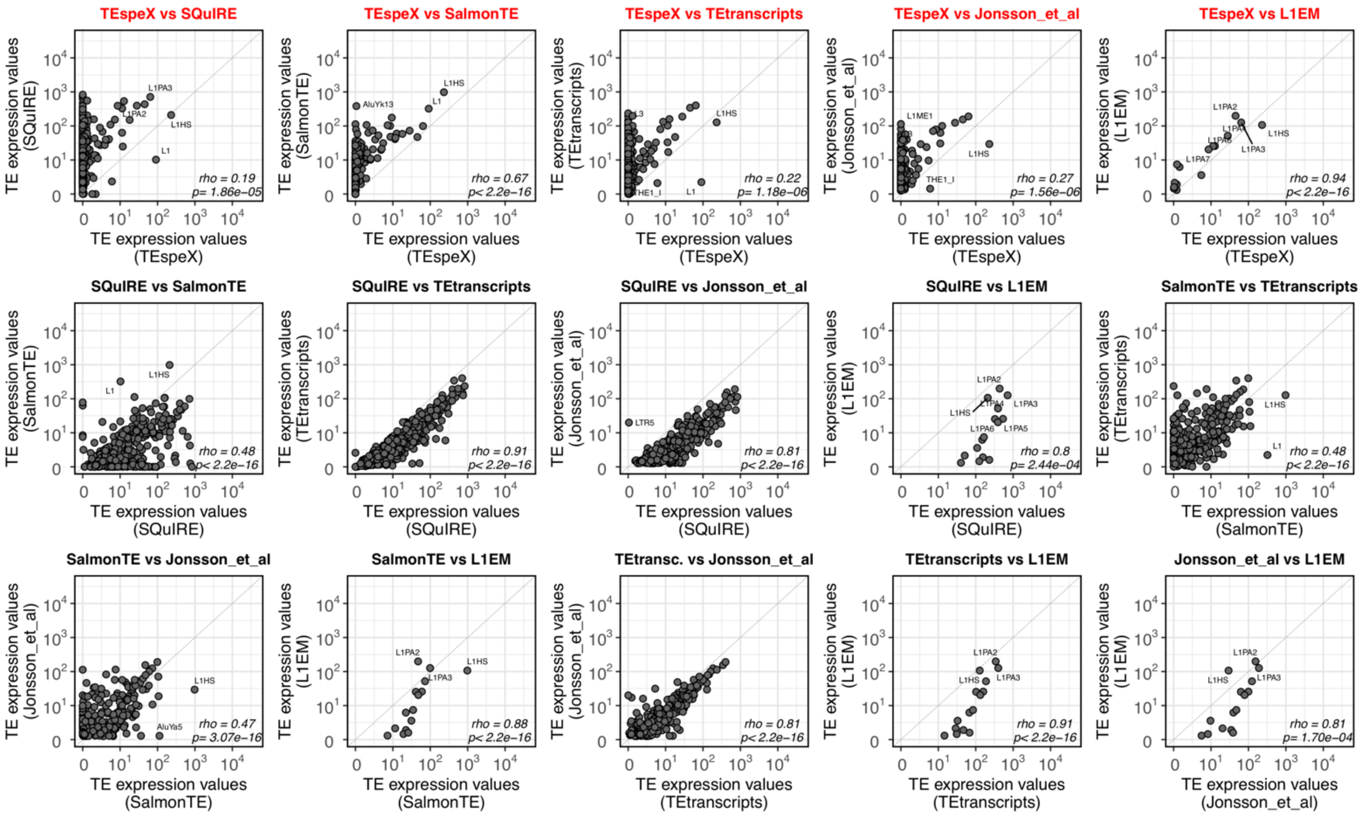

Supplementary Figure S7: comparison among different TE expression quantification standalone pipelines

Scatter plots reporting the TE expression levels calculated by the different tools tested. Each dot represents the mean TE expression levels calculated among the entire Jönsson *et al.* dataset. Spearman's rank correlation coefficients (rho) and p-values are reported for each comparison. Red-colored titles indicated comparisons involving TEspeX.

Supplementary Data Note 8

Differentially expressed TEs in the Jönsson *et al.* dataset – TEspeX analysis

Differentially expressed TEs in hNPC upon the KO of DNMT1 were identified as described in **Supplementary Data Note 6**, except for the log2 fold-change threshold used to call a TE as differentially expressed that was removed, following the approach used by the authors (*i.e.,* a given TE was considered as differentially expressed when showing FDR<0.05, regardless of its fold-change value).

Our results highlighted 8 TEs significantly up-regulated upon the KO of DNMT1 whereas no down-regulated TEs were observed (**Supplementary Fig. S8**). Of the 8 up-regulated TEs, 7 were young LINE-1 elements and the remaining one an LTR element (LTR12C). Notably, these results are in high concordance with the data described by the authors of the article which observed that the majority of the significantly up-regulated TE loci belonged to young LINE-1 families (L1HS, L1PA2 and L1PA3 subfamilies) as well as to the LTR family LTR12C.

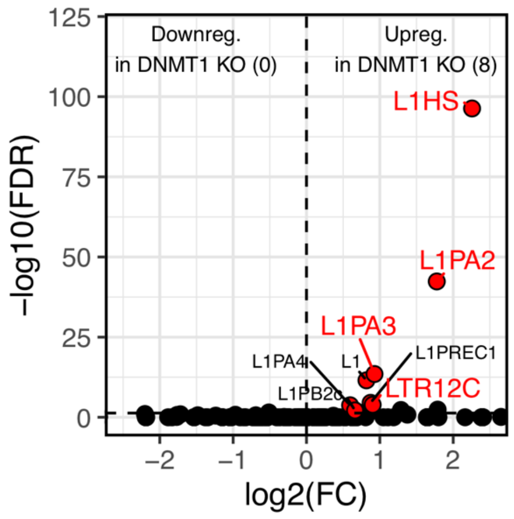

Supplementary Figure S8: differentially expressed TEs upon DNMT1 KO.
TE expression levels were calculated by TEspeX in control and DNMT1 KO hNPCs. Identification of the differentially expressed TEs performed by edgeR highlighted 8 up-regulated TEs while no down-regulated TE was observed. The red-colored TEs are those resulting as strongly differentially expressed by the authors of the paper.

Supplementary Data Note 9

Differentially expressed TEs in the Jönsson *et al.* dataset – example of TEspeX using intron retention analysis correction

Intron sequences are usually not part of mature mRNAs, however, it may happen that some introns are unexpectedly retained during the sequencing library preparation or that they are actively retained in specific biological contexts (intron retention, referred as IR). To define whether the potential retention of introns might be a source of TE passive expression, the intron retained in each sample of the Jönsson *et al.* dataset (Jönsson *et al.*, 2019) were identified and the TEspeX analysis repeated filtering-out the sequencing reads mapping to the identified retained introns.

Retained introns were identified by using IRFinder (Middleton *et al.*, 2017), discarding introns in overlap with other features (such as an alternative exons) and considering a given intron as retained if the intron depth is >=3, intron retention (IR) ratio is >=0.1 and no warnings were generated during the analysis.

By applying these filters, a total number of 3,055 introns resulted retained among the six samples analysed. In particular, between 908 and 1,257 introns resulted retained in each sample (**Supplementary Fig. S9A**). IR appeared to be a sample-specific feature, with most of the introns being retained just in one sample (**Supplementary Fig. S9B**). Indeed, only 102 of the 3,055 retained introns resulted commonly retained in all the analysed samples (**Supplementary Fig. S9B**). Next, the TE expression levels were recalculated by TEspeX discarding, in addition to the reads mapping on coding/non-coding transcripts, the reads mapping on the retained introns. To this end, the nucleotide sequences of the introns resulting retained in each specific sample were added to the sequences of the coding/non-coding transcripts by using the TEspeX *--mask* parameter. The TE expression levels were then

calculated in each sample. Our results highlighted that discarding or not discarding RNA-seq reads mapping to retained introns slightly affects the overall TE expression levels (**Supplementary Fig. S9C**). In particular, when sequencing reads mapping to retained introns were discarded, only 6 TE consensus changed their expression levels in at least one analysed sample by more than 0.2-fold compared to the default TEspeX analysis (**Supplementary Fig. S9C**). As a consequence of this, when the differentially expressed TEs analyses was repeated, no differences were found between the set of TEs resulting DE in the default TEspeX analysis and those resulting DE in the TEspeX analysis where RNA-seq reads mapping to retained introns were discarded (**Supplementary Fig. S9D**). The expression levels of the DE TEs were indeed identical between the two analyses (**Supplementary Fig. S9E**).

Altogether, these results suggest that, in the analysed dataset, retained introns do not significantly contribute to the generation of TEs passive expression. The performed correction however can results useful in cases in which differential intron retention or any bias given by intronic reads is present in the analyzed samples.

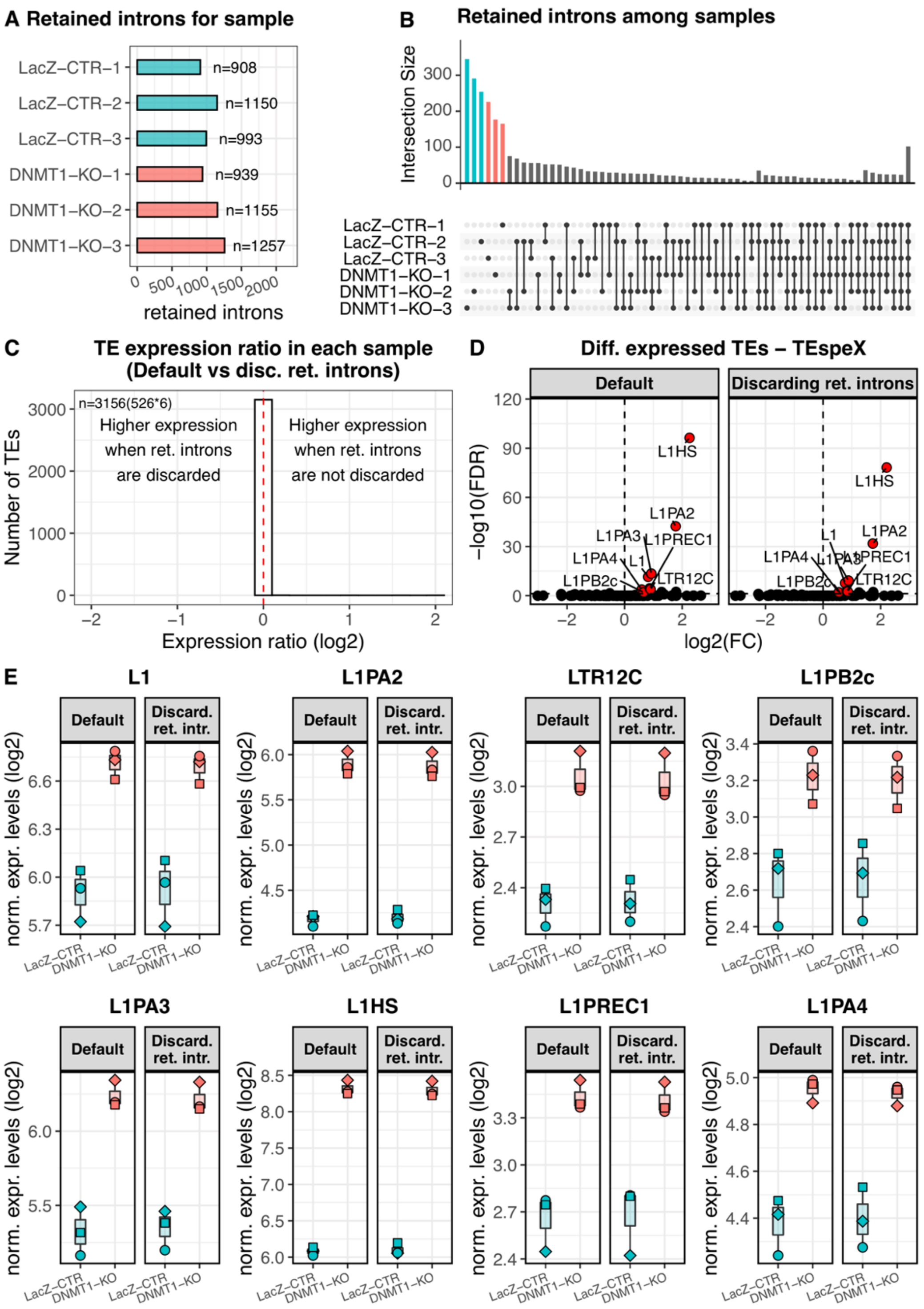

Supplementary Figure S9: TEspeX analysis on Jönsson *et al.* dataset, discarding RNA-seq reads mapping to retained introns.

(**A**) Number of retained introns per each analysed sample. (**B**) Upset plot showing the number of introns that are retained in each specific sample and among the different samples. (**C**) Distribution of the TE expression ratio in the default TEspeX analysis and in the TEspeX analysis where RNA-seq reads mapping to retained introns were discarded. Ratio has been calculated dividing the TE expression values calculated in the default TEspeX analysis by the TE expression levels calculated discarding all the RNA-seq reads mapping to retained introns. This has been done for each TE consensus analysed (526) in all the analysed samples (6). Log2 of the ratio is represented. Bars width is set to 0.2. (**D**) Volcano plot showing the differentially expressed TEs in the default analysis (left, the same as in **Supplementary Figure S8**) and in the analysis where RNA-seq reads mapping to retained introns were discarded. (**E**) Expression profiles of the DE TE consensus.

Supplementary Data Note 10

Testing TEspeX on evolutionary old and young TEs

An additional validation of the capability of TEspeX in measuring authentic TE transcription, is to test the tool on a set of evolutionary young and old TEs. Theoretically, evolutionary old TEs, unlike the young ones, should not result expressed as more likely to have accumulated genomic alterations impairing their transcription.

To this end, all the transposable elements annotated in the *Drosophila* (dm6) and *H. sapiens* (hg38) genomes were retrieved from the Repeatmasker database (Smith, AFA *et al.*, 2013) discarding all the TEs not annotated as 'DNA', 'LINE', 'LTR', 'Retroposon' and 'SINE'. The TE loci were next classified according to: i) their localisation respective to the genic features (genic, intronic and intergenic) and ii) their evolutionary age that was calculated based on the number of mismatches carried by each TE locus (milliDiv score). Next, the 10 youngest and the 10 oldest intergenic and intronic TE loci were selected for both *Drosophila* and *H. sapiens* (note that only one TE for each subfamily has been selected. Therefore, if more than one TE belonging to the same subfamily was listed in the top 10, only the first was selected). The specific selected TE loci sequences were then added to the TE consensus fasta file of each species and, finally, the expression of all the TEs was calculated by TEspeX from the *Drosophila* and *H. sapiens* RNA-seq datasets previously analyzed (Jönsson *et al.*, 2019; Krug *et al.*, 2017) using only the control samples.

Our results showed that TEspeX successfully detected the expression of young TEs, for both the intergenic and intronic ones, in both *Drosophila* and *H. sapiens* (**Supplementary Fig. S10**). On the contrary, the expression of the old TEs was overall not detected, for both TE types (*i.e.*, intergenic and intronic) and species (**Supplementary Fig. S10**). However, while the expression of the old TEs in human was null for all the TE analysed, a few TEs showed a very low level of

expression in *Drosophila*. Although this evidence of transcription is really low and might depend on several factors, a possible explanation could be associated to the evolutionary processes that have characterised the TE evolution within the fly genome. While in humans only a couple of young elements are actually transcribed (*e.g.*, L1HS, L1P, AluY, ERV), in *Drosophila* a much greater diversity of TEs is transcribed and the separation between young and old elements is less strict than in humans. Therefore, some TEs can potentially be transcribed, although having been classified as evolutionary old in our analysis.

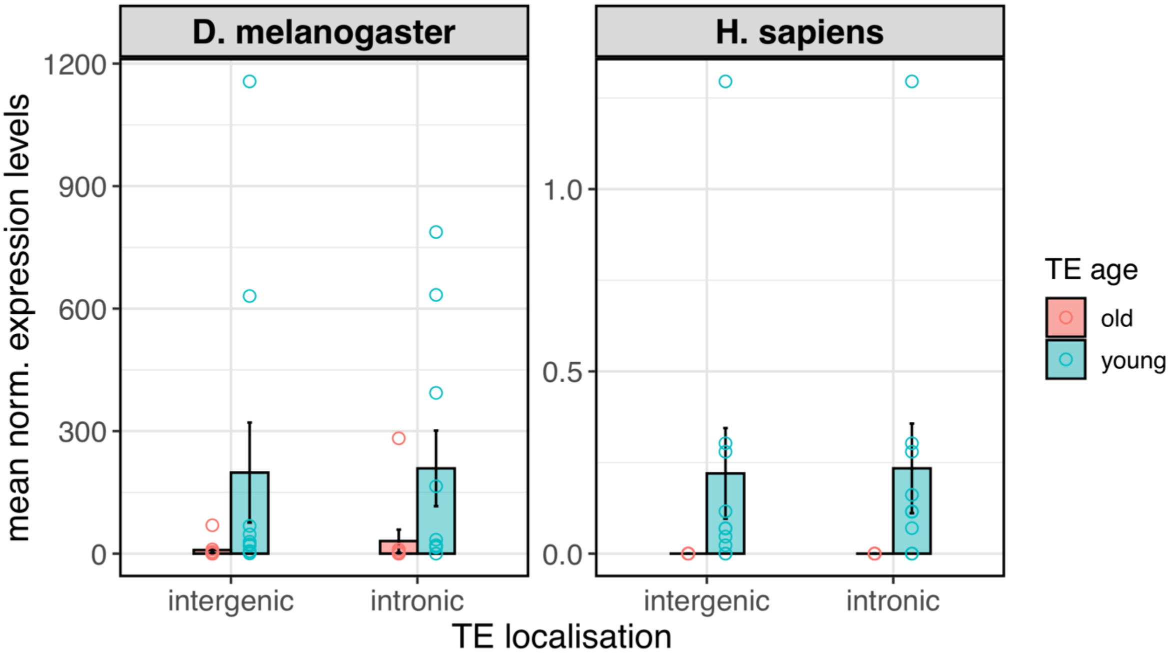

Supplementary Figure S10: expression of old and young TEs.
The expression of old (red) and young (blue) TEs were tested by TEspeX on the *Drosophila* and *H. sapiens* RNA-seq datasets previously used (Jönsson *et al.*, 2019; Krug *et al.*, 2017). Each data point indicates the mean of expression of each analysed TEs among the different biological replicates. Barplots indicate the mean of expression (+/- sem) calculated between the 10 TEs analysed in each group. While the young TEs resulted expressed (in both intronic/intergenic, in both species) the old TEs resulted, overall, not expressed.

Supplementary Data Note 11

Defining the fraction of each TE consensus displaying a perfect homology with non-TE transcripts

TEspeX is implemented to discard the sequencing reads potentially transcribed from TE fragments embedded in non-TE transcript. To this end, all the reads aligning with best alignment score to non-TE transcripts are discarded. However, when a perfect sequence homology between a given portion of a TE consensus sequence and a non-TE transcripts exists, TEspeX cannot unambiguously assign the reads that are consequently discarded from the analysis. To define how frequently this occurs, every *Drosophila* and *H. sapiens* TE consensus sequence was split in overlapping *k-mers* of 50 nucleotides with 1 nucleotide step. Each *k-mer* was then aligned to the annotated coding and non-coding transcripts by using blast (*blastn*) (Altschul *et al.*, 1990), retrieving only the perfect and complete matches *(-word_size* 50 -*perc_identity* 100). The matches representing the same portion of each TE consensus were collapsed by using bedtools merge (Quinlan and Hall, 2010). This allowed to estimate the portion of each TE consensus that displays a perfect homology, of at least 50 nucleotides, with at least one canonical transcript.

Reassuringly, our results highlighted that the majority of the TE consensus of both *Drosophila* and *H. sapiens* did not display regions of perfect homology of at least 50 nucleotides with canonical transcripts (**Supplementary Fig. S11**). In particular, this was observed for 202 of the 247 (82%) *Drosophila* TE consensus analysed and for 357 of the 526 (68%) human TE consensus analysed (**Supplementary Fig. S11**). On the other hand, only three TE consensus in each of the two species showed a 100% homology with canonical non-TE transcripts. Namely, these three TE consensus are: BURDOCK_LTR, ROO_LTR and STALKER2_LTR (*Drosophila*) and AluYa5, AluYb8 and AluYb9 (*H. sapiens*). According to these results, TEspeX might be inefficient in calculating the expression levels for these 6 TEs. However, modern sequencing technologies

currently produce paired-end sequencing reads of at least 100 nucleotides per pair (100x2) which might facilitate the resolution of such ambiguous situations. Indeed, when repeating the same analysis generating from TE consensus *k-mers* of 200 nucleotides, instead of 50, no *Drosophila* TE showed a 100% homology with any non-TE transcripts whereas only two human TEs (AluYa5 and AluYb8) still showed a complete homology with canonical transcripts (data not shown). However, both TE consensus result entirely contained in different annotated transcripts thus revealing that, by using short read sequencing methods, an unambiguous assignment of the reads deriving from the expression of these two TEs is not possible for any existing tool.

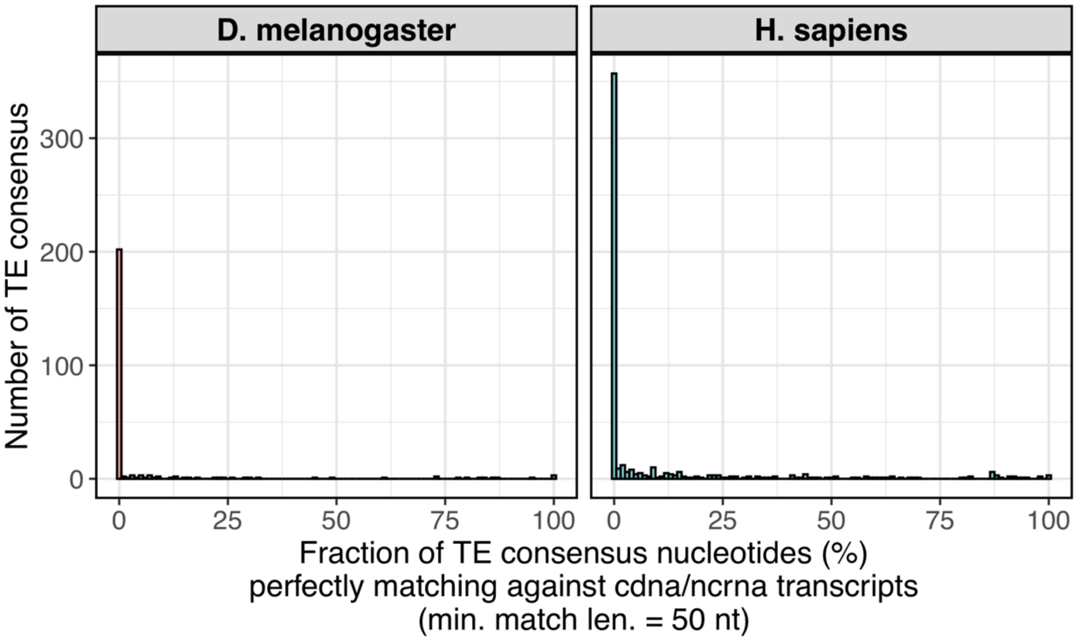

Supplementary Figure S11: percentage of nucleotides of each TE consensus displaying perfect homology with canonical transcripts.
Histograms reporting the distributions of the percentages of nucleotide of each *D. melanogaster* and *H. sapiens* TE consensus displaying a perfect sequence homology with canonical non-TE transcripts. In both the analysed species, the majority of the TE consensus (202/247 in *Drosophila* and 357/526 in *H. sapiens*) show no regions of perfect homology, of at least 50 nucleotides, with canonical non-TE transcripts.

Supplementary Data Note 12

Comparison between RAMPAGE and TEspeX in measuring *Alu* expression

RAMPAGE technique (Batut and Gingeras, 2013) is a 5′-complete cDNA sequencing assay that captures the transcription start site (TSS) at single-nucleotide resolution. This allows to identify with extreme sensitivity the TSS of a given transcribed feature and to quantify its expression level. In 2019, Zhang and colleagues exploited the RAMPAGE technique to identify the TSS of Pol-III transcribed *Alu* and to quantify their expression levels genome-wide (Zhang *et al.*, 2019). Moreover, Zhang and colleagues also performed RNA-seq from the very same samples sequenced by RAMPAGE. This allowed us to calculate the *Alu* expression levels in the RNA-seq data by using TEspeX, SalmonTE, TEtranscripts and SQuIRE and to compare the calculated expression levels with the ones calculated by Zhang and colleagues using the RAMPAGE technique.

To this end, first, the normalised expression levels (RPM) of the *Alu* RAMPAGE peaks were retrieved from the supplementary table S2 of the Zhang and colleagues' manuscript (Zhang *et al.*, 2019) for three arbitrarily selected samples (suprapubic skin female adult 53 years old, thoracic aorta male adult 37 years old, tibial nerve female adult 53 years old). Second, the TE expression levels in the respective RNA-seq data were calculated by TEspeX, SalmonTE, TEtranscripts and SQuIRE (as described in **Supplementary Data Note 3**). Third, Spearman correlation was used to calculate the degree of correlation between the TE expression levels calculated by the different tools. All the analyses were done at the TE consensus level. For locus-specific tools/techniques (RAMPAGE and SQuIRE) expression levels of single loci belonging to the same *Alu* subfamily were summed, following the same workflow described in **Supplementary Data Note 3**.

Our results showed no statistically significant correlation between the *Alu* expression values calculated by TEspeX and those calculated by RAMPAGE, with many Alu resulting not expressed according to TEspeX and showing evidence of transcription according to RAMPAGE, in all the analysed datasets (**Supplementary Fig. S12**). Similarly, no significant correlation was shown between the expression levels calculated by SalmonTE and by RAMPAGE (**Supplementary Fig. S12**). On the contrary, TEtranscripts and SQuIRE resulted capable to quantify *Alu* expression showing positive and significant correlation with RAMPAGE (**Supplementary Fig. S12**) (rho >= 0.95 and p-value < 2.2e-16 for both tools, in all the analysed datasets) (**Supplementary Fig. S12**). Of note, both TEtranscripts and SQuIRE showed the tendency to overestimate the *Alu* expression compared to RAMPAGE (**Supplementary Fig. S12**).

These results suggested that TEspeX and SalmonTE cannot properly estimate the expression values of some human *Alu*. For TEspeX this is likely the consequence of the fact that *Alu* have been very frequently exonized (Schmitz and Brosius, 2011). Consequently, RNA-seq reads deriving from the transcription of exonized *Alu* can be mapped to both TE and non-TE transcripts. In these ambiguous situations, it is not possible to discriminate whether the RNA-seq reads indicate autonomous or passive expression and the reads are therefore discarded by TEspeX. Our choice has been to be very stringent for the elements for which the annotated exonized sequence is identical to that of an autonomous copy even if this leads to the generation of false negatives. More analyses are instead needed to understand the reason of the lack of correlation relatively to SalmonTE. Finally, the strong and general overestimation of the expression generated by TEtranscripts and SQuIRE is likely caused by the addition of reads deriving by exonized *Alu* to the reads deriving by their autonomous transcription.

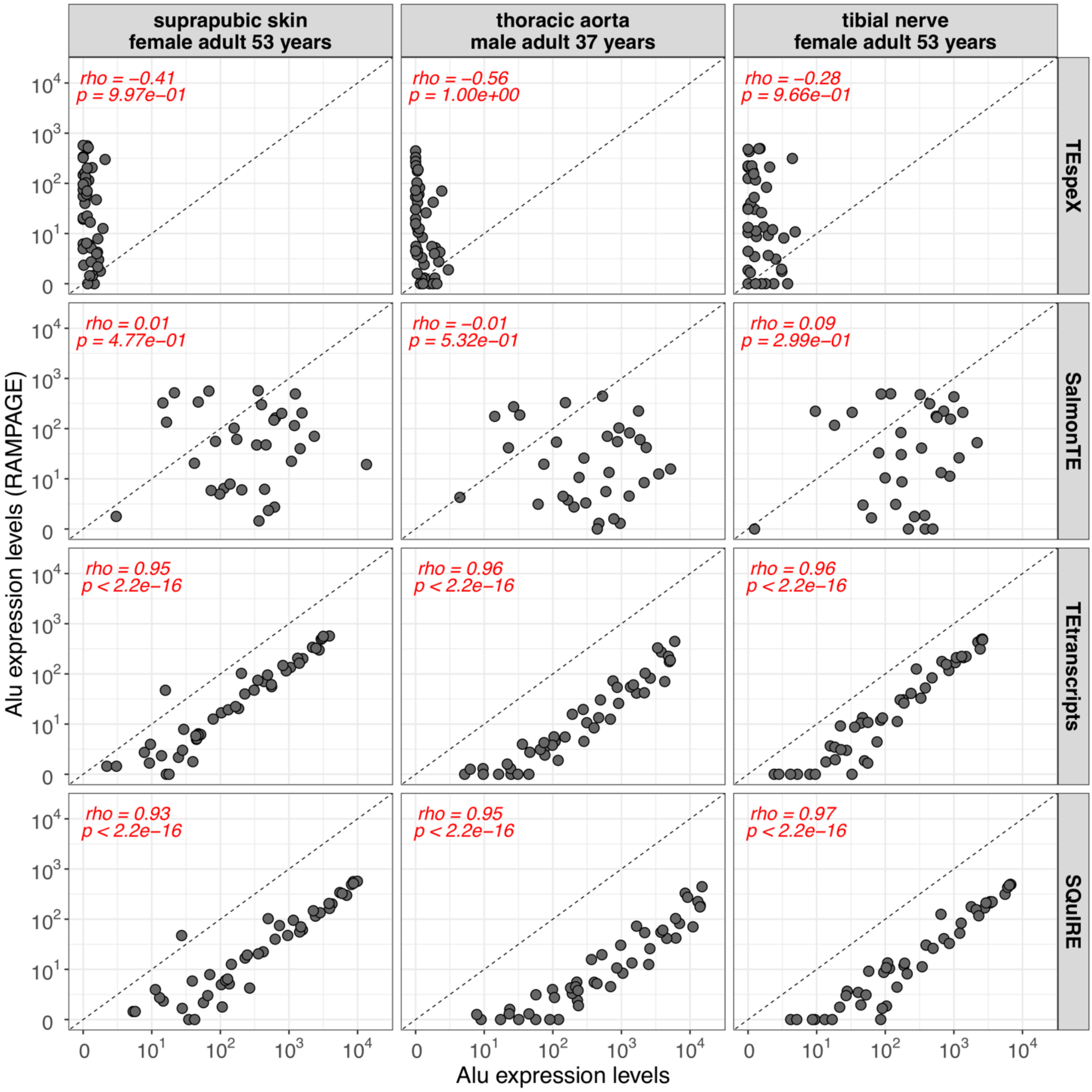

Supplementary Figure S12: correlation between TE expression levels calculated by RAMPAGE, TEspeX, SalmonTE, TEtranscripts and SQuIRE.

Scatter plots showing the correlation between TE expression levels calculated by RAMPAGE (y-axis) and TEspeX, SalmonTE, TEtranscripts and SQuIRE (x-axis). Spearman's rank correlation coefficients (rho) and p-values are reported for each comparison.

Supplementary references

Altschul,S.F. *et al.* (1990) Basic local alignment search tool. *J. Mol. Biol.*, **215**, 403–410.

Bao,W. *et al.* (2015) Repbase Update, a database of repetitive elements in eukaryotic genomes. *Mob. DNA*, **6**, 11.

Batut,P. and Gingeras,T.R. (2013) RAMPAGE: promoter activity profiling by paired-end sequencing of 5'-complete cDNAs. *Curr. Protoc. Mol. Biol.*, **104**, Unit 25B.11.

Bushnell,B. (2014) BBMap. *https://sourceforge.net/projects/bbmap/*.

Dobin,A. *et al.* (2013) STAR: ultrafast universal RNA-seq aligner. *Bioinforma. Oxf. Engl.*, **29**, 15–21.

Farré,D. *et al.* (2016) Novel Role of 3'UTR-Embedded Alu Elements as Facilitators of Processed Pseudogene Genesis and Host Gene Capture by Viral Genomes. *PloS One*, **11**, e0169196.

Frazee,A.C. *et al.* (2015) Polyester: simulating RNA-seq datasets with differential transcript expression. *Bioinformatics*, **31**, 2778–2784.

Hubley,R. *et al.* (2016) The Dfam database of repetitive DNA families. *Nucleic Acids Res.*, **44**, D81-89.

Jeong,H.-H. *et al.* (2018) An ultra-fast and scalable quantification pipeline for transposable elements from next generation sequencing data. *Pac. Symp. Biocomput. Pac. Symp. Biocomput.*, **23**, 168–179.

Jin,Y. *et al.* (2015) TEtranscripts: a package for including transposable elements in differential expression analysis of RNA-seq datasets. *Bioinforma. Oxf. Engl.*, **31**, 3593–3599.

Jönsson,M.E. *et al.* (2019) Activation of neuronal genes via LINE-1 elements upon global DNA demethylation in human neural progenitors. *Nat. Commun.*, **10**, 3182.

Krug,L. *et al.* (2017) Retrotransposon activation contributes to neurodegeneration in a Drosophila TDP-43 model of ALS. *PLOS Genet.*, **13**, e1006635.

Li,H. *et al.* (2009) The Sequence Alignment/Map format and SAMtools. *Bioinforma. Oxf. Engl.*, **25**, 2078–2079.

Li,H. and Durbin,R. (2009) Fast and accurate short read alignment with Burrows-Wheeler transform. *Bioinforma. Oxf. Engl.*, **25**, 1754–1760.

McKerrow,W. and Fenyö,D. (2019) L1EM: a tool for accurate locus specific LINE-1 RNA quantification. *Bioinformatics*, btz724.

Middleton,R. *et al.* (2017) IRFinder: assessing the impact of intron retention on mammalian gene expression. *Genome Biol.*, **18**, 51.

Quinlan,A.R. and Hall,I.M. (2010) BEDTools: a flexible suite of utilities for comparing genomic features. *Bioinformatics*, **26**, 841–842.

Robinson,M.D. *et al.* (2010) edgeR: a Bioconductor package for differential expression analysis of digital gene expression data. *Bioinformatics*, **26**, 139–140.

Schmitz,J. and Brosius,J. (2011) Exonization of transposed elements: A challenge and opportunity for evolution. *Biochimie*, **93**, 1928–1934.

Smith, AFA *et al.* (2013) RepeatMasker Open-4.0.

Yang,W.R. *et al.* (2019) SQuIRE reveals locus-specific regulation of interspersed repeat expression. *Nucleic Acids Res.*, **47**, e27.

Zerbino,D.R. *et al.* (2018) Ensembl 2018. *Nucleic Acids Res.*, **46**, D754–D761.

Zhang,X.-O. *et al.* (2019) Genome-wide analysis of polymerase III-transcribed Alu elements suggests cell-type-specific enhancer function. *Genome Res.*, **29**, 1402–1414.
